# Is Laryngeal Squamous Cell Carcinoma Related to *Helicobacter pylori?*

**DOI:** 10.3389/fonc.2022.790997

**Published:** 2022-01-28

**Authors:** Yan Huang, Min Gu, Qi Wu, Juanfen Zhu, Jian Wu, Peipei Wang, Meihua Wang, Judong Luo

**Affiliations:** ^1^Department of Radiotherapy, The Affiliated Changzhou No.2 People’s Hospital of Nanjing Medical University, Changzhou, China; ^2^Department of Head and Neck Surgery, Graduate School of Dalian Medical University, Dalian, China; ^3^Department of Stomatology, Affiliated Third Hospital of Soochow University, The First People’s Hospital of Changzhou City, Changzhou, China; ^4^Department of Clinical Medicine, Heze Medical College, Heze, China; ^5^Department of Head and Neck Surgery, The Affiliated Changzhou No.2 People’s Hospital of Nanjing Medical University, Changzhou, China; ^6^Department of Pathology, Changzhou Tumor Hospital, Affiliated to Soochow University, Changzhou, China

**Keywords:** laryngeal malignancy, *Helicobacter pylori*, laryngopharyngeal reflux, prognosis, cancer

## Abstract

**Background:**

Laryngeal carcinoma is a primary malignant tumor originating from the laryngeal mucosa. In recent years, an increasing number of studies have confirmed that Helicobacter pylori may play a role in the occurrence and development of laryngeal cancer. We conducted a systematic review and meta-analysis to identify and emphasize the relationship between laryngeal cancer and Helicobacter pylori infection.

**Methods:**

We actively searched for systematic reviews of PubMed, Embase, Web of Science, and Cochrane libraries to select studies that met the recommended guidelines. A total of 1756 patients with laryngeal cancer were included in this study to assess the association of *Helicobacter pylori* in the larynx with laryngeal cancer. A subassessment of the risk of bias for each study that met the inclusion criteria was carried out. To illustrate the reasons for heterogeneity, we performed a subgroup analysis to determine the type of study, the quality of the article, the diagnostic method, and the impact of exposure factors.

**Results:**

The meta-analysis included a total of 17 case-control studies on the association between Helicobacter pylori in the larynx and laryngeal cancer. Our meta-analysis showed that Helicobacter pylori infection in the larynx significantly increased the risk of malignant tumors in the larynx (odds ratio, 2.96; 95% CI, 1.83-4.78; P<0.00001; I^2 =^ 86%). They still existed when we controlled for patients’ exposure to smoking factors (odds ratio, 3.86; 95% CI, 1.89–7.88).

**Conclusion:**

This systematic review and meta-analysis showed an association between Helicobacter pylori and laryngeal cancer. These findings are consistent with the understanding that chronic inflammatory tissue insult may lead to the development of malignancy. Controlling risk factors will help us identify patients with an increased risk of laryngeal cancer.

## Introduction

Laryngeal cancer is the 14th most common cancer in the 7th quarter of the world, with hundreds of thousands of new cases every year (1). When these cancers are detected by a doctor, they are usually in advanced state (2). The destructive nature of cancer suggests that the 5-year overall survival rate is poor, and the early survival rate is higher (3). To improve the detection rate, we need to understand the causal factors of this cancer. This will significantly enhance the efficiency of clinical work. Tobacco and alcohol are closely related factors of laryngeal cancer. Although tumors can develop anywhere in the throat, the glottis is the most common site, followed by the supra-glottis and the sub-glottis (4). With the increase in daily alcohol consumption, the risk of laryngeal cancer increased sharply, and there was no apparent threshold effects at lower intensities (5). Through the prevention and control of tobacco use and comprehensive cancer control efforts, the incidence of tobacco-related cancers, such as laryngeal cancer, can be reduced (6). Ten symptoms are significantly related to laryngeal cancer. The single biggest risk factor is hoarseness. Other risk markers included sore throat, earache, and insomnia (7).HNSCC in general are usually diagnosed in advanced tumor stage, however glottis laryngeal carcinoma can be diagnosed earlier due to the early complaints of hoarseness. HPV infection, especially infection caused by high-risk HPV-16, is significantly related to the incidence of laryngeal cancer (8). Convenient and efficient HPV detection methods can effectively prevent laryngeal cancer and control its development (9). Other risk factors may also play a significant role in the development of laryngeal squamous cell carcinoma. The published literature suggests that reflux diseases, such as gastroesophageal reflux disease and laryngopharyngeal reflux, have a significant relationship with malignant tumors of the larynx (10).

*Helicobacter pylori* colonizes the human gastric mucosa and is associated with specific diseases. Virulence factors, such as urease, vacuolar toxin (VacA), and cytotoxin-associated antigen CagA, may lead to different diseases development (11). *Helicobacter pylori* has a mechanism for reproduction. It can survive in very acidic ecological niches (12). Urease-catalyzed hydrolysis of urea produces ammonia (NH3) and carbamate. In this way, the abundance of NH 3 and CO 2 provided a specific environment for the survival of *Helicobacter pylori (*13, 14). *Helicobacter pylori* plays a role in the disease progression of gastric cancer and mucosa-associated lymphoid tissue lymphoma (15). Presumably, laryngeal reflux and gastroesophageal reflux disease provide an acidic environment for the larynx, according to the work of Sean M. Parsel et al. (10). Here, part of *Helicobacter pylori* can be identified and lead to subsequent cancer development and progression. Many studies have indicated that H. pylori may produce a marked effect in the development of laryngeal cancer. For example, it can be detected in normal people’s larynx, benign vocal cord tumors, and vocal cord leukoplakia (16–18). In addition, *Helicobacter pylori* could induce systemic inflammation and promote the occurrence of tumors (19). Considering the potential connection between *Helicobacter pylori* infection and the development of malignant tumors, the purpose of this study was to investigate the relationship between *Helicobacter pylori* and laryngeal cancer and to clarify the impact of *Helicobacter pylori* infection on the development of laryngeal cancer.

## Methods

### Literature Search Strategy

A comprehensive search was performed in the PubMed, EMBASE, Cochrane Library, and Web of Science databases from their inception through July 5, 2021. The search criteria included all occurrences in the title or abstract of the following terms: *Helicobacter pylori* plus laryngeal cancer or laryngeal malignancy. The specific search item was “(Helicobacter pylori OR Helicobacter nemestrinae OR Campylobacter pylori OR Campylobacter pylori subsp. pylori OR Campylobacter pyloridis) AND (Laryngeal Neoplasms OR Neoplasms, Laryngeal OR Laryngeal Neoplasm OR Neoplasm, Laryngeal OR Larynx Neoplasms OR Larynx Neoplasm OR Neoplasm, Larynx OR Neoplasms, Larynx OR Cancer of Larynx OR Larynx Cancers OR Laryngeal Cancer OR Cancer, Laryngeal OR Cancers, Laryngeal OR Laryngeal Cancers OR Larynx Cancer OR Cancer, Larynx OR Cancer of the Larynx)”. The study population included adult males and females. The intervention was the detection of *Helicobacter pylori* infection in the larynx, and the control was a patient without *Helicobacter pylori* infection. The result was a control evaluation of patients previously diagnosed with *Helicobacter pylori* infection to obtain an odds ratio (OR) of 95% CI associated with laryngeal cancer. The study design included case-control and cohort studies and randomized controlled trials. In addition to malignant tumors of the larynx, a literature search was conducted on studies assessing the risk of esophageal cancer and gastric cancer to obtain more citations for review. The preferred reporting item criteria for systematic reviews and meta-analysis were used to ensure that the systematic reviews were adequately reported.

### Selection Criteria

Two reviewers (YH and MG) independently assessed the qualifications of the data in a standardized manner. The retrieved duplicate records have been deleted. The abstract of each citation was then screened and evaluated for adult *Helicobacter pylori* infection and its relationship with malignant tumors of the larynx. Irrelevant citations, such as case reports and studies without a control group, were excluded. However, full text of the remaining citations and other records from the list of references in published articles were included. Full papers were carefully reviewed, and studies that did not meet the criteria were excluded. Studies that were not written in English and did not provide patients’ demographic characteristics, site of malignancy, diagnostic criteria, or quantitative data on patients were excluded. Articles related to benign laryngeal disease were excluded. Studies on respiratory and digestive malignancies excluding the larynx were not included. The remaining studies were included in this analysis.

### Data Extraction

To ensure data extraction accuracy, the two authors extracted data according to the inclusion and exclusion criteria, and the third author assisted in making the final decision on disputed information. The extracted data included the author’s name, publication year, country, tumor type, the total number of patients and detection methods, and history of malignant tumors of the larynx. The primary summary measure was exposure to *Helicobacter pylori* and subsequent laryngeal cancer development. The raw data of the number of exposed and unexposed patients and the number of patients with or without disease were extracted for quantitative meta-analysis. The risk of bias in the article was roughly assessed by checking the design of each study and the proposed research purpose.

### Quality Assessment

Methodological quality was systematically reviewed and meta-analyzed based on the National Heart, Lung and Blood Institute (NHLBI). It is a widely accepted and valid tool for observational and nonrandomized studies in the surgical profession. This tool was used for a semiquantitative assessment of the quality of nonrandomized studies. Based on the opinions of the two reviewers, each study was subjectively rated as having a “high”, “moderate”, or “low” risk of bias rating (**Table 1**).

**Table 1:** Quality assessment of the selected studies for meta-analysis.

S. no	Criteria	High	Moderate	Low
1	Purpose of this study	17	—	—
2	Eligibility criteria	14	2	1
3	Sample size adjustment	17	—	—
4	Research group of people	17	—	—
5	Range of anatomical parts	17	—	—
6	Definition of the measurement used	17	—	—
7	Outcome measures(OR.95%CI)	14	—	3

### Statistical Methods

The Mantel-Haenszel method with random effects was used for meta-analysis. Use I^2^ statistics to assess heterogeneity. In all included studies, the association between *Helicobacter pylori* and malignant tumors was evaluated by the OR of 95% CI. An I^2^> 50% was considered heterogeneous. The source of heterogeneity was studied through sensitivity analysis. Furthermore, the study design (retrospective or prospective), diagnostic methods, methodological quality, and exposure risk factors’ influence on the overall OR effect were carried out. A funnel plot was used to assess publication bias and conduct sensitivity analysis to determine the impact of individual studies.Stata 15.1 and Review Manager 5.2 software were used for analysis.

### Publication Bias

All included studies used Egger’s bias assessment graph test to construct funnel plots (scatter plots constructed using standard error [Y axis] and Log (HR)[X axis]). The symmetry of the study distribution on the regression line is inversely proportional to the size of the publication bias in the meta-analysis.In the process of data selection and quality assessment, any differences are resolved through discussion among the reviewers.

## Results

### Study Selection

**Figure 1:**
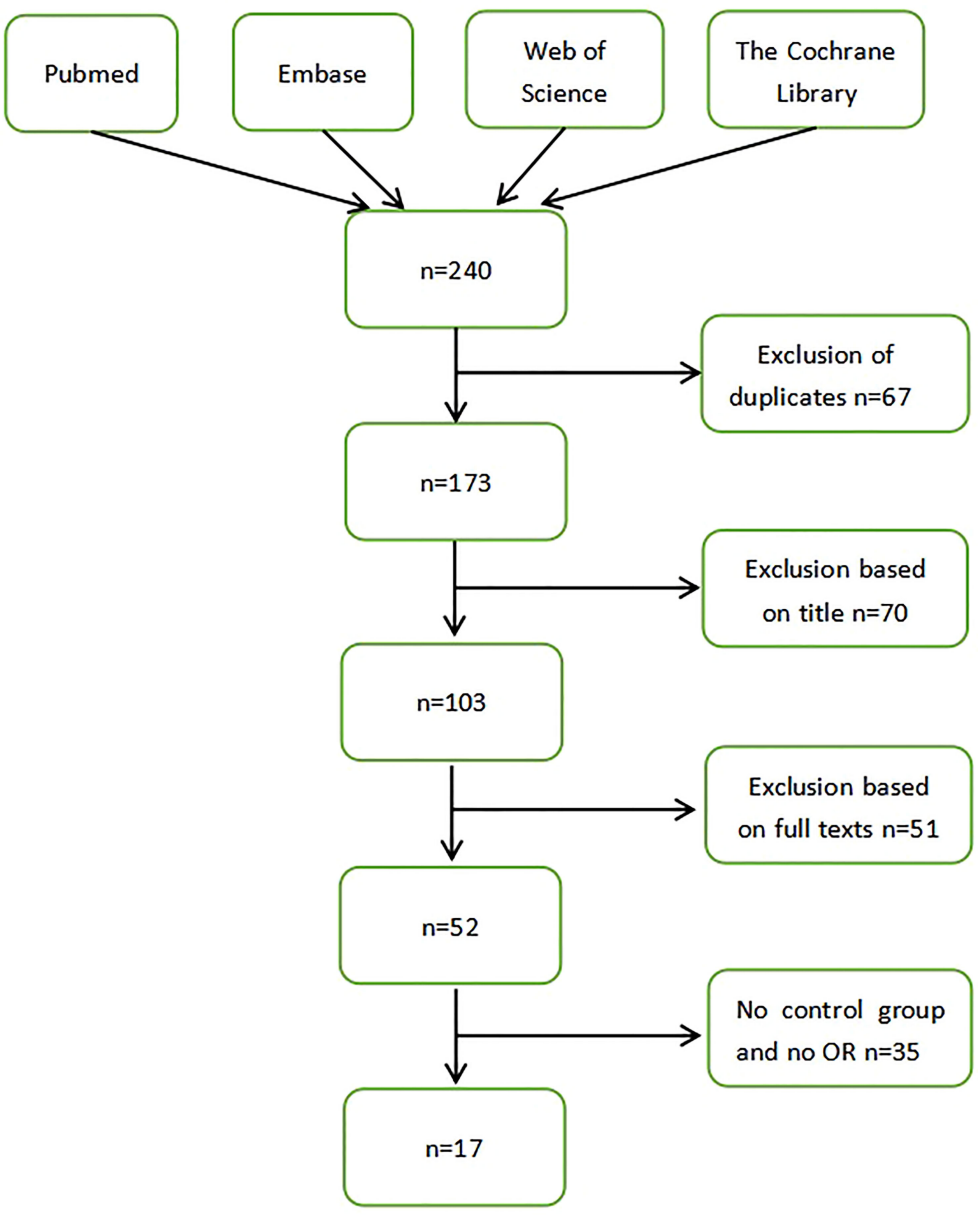
Flow diagram showing study inclusion and exclusion.

A total of 240 articles were identified by searching the database. We selected according to the correlation between *Helicobacter pylori* and the progression of malignant tumors of the larynx. After importing document management software to delete duplicate records, 173 articles were considered for review of titles and abstracts. Among them, 70 studies were excluded from the analysis of article titles and abstracts. A total of 103 articles were collected for full-text review. Due to the lack of a control group in the experimental design, 35 studies were excluded. A total of 17 articles were included for this systematic review and meta-analysis. There was a trend of a positive correlation between Helicobacter pylori infection and malignant tumors of the larynx (**Figure 1**).

### Study Characteristics

**Table 2** describes the study design and patient information for all 17 studies. All 17 included studies adopted a case-control design, of which 4 were retrospective studies, and 13 were prospective data. Based on the National Heart, Lung and Blood Institute (NHLBI), quality assessment template, a systematic review and meta-analysis of the method quality were carried out.

**Table 2:** Characteristics of the included studies of the meta-analysis.

Author	Year	Case	Assay Method	Country	Gender	Age	Research method	Risk Factor	Quality assessment
Amizadeh, M.et al (20)	2014	144	PCR	Iran	Men118 Women26	Mean 52.6	Retrospective research	None	High
Aygenc, E.et al (21)	2001	58	ELISA	Turkey	Unknown	Mean 56.7	Prospective research	None	Moderate
Barakat, G.et al (22)	2016	49	PCR	Egyp	Unknown	Mean 66.1	Prospective research	None	Moderate
Chen, M. et al (18)	2019	41	PCR	China	Men 40 Women 1	Mean 58.1	Prospective research	None	High
Fellmann, J.et al (16)	2013	8	PCR	Switzerland	Men 3 Women 5	Mean 50	Retrospective research	Smoke	Low
Genç, R.et al (23)	2013	59	ELISA	Turkey	Unknown	Mean 50.6	Prospective research	None	High
Gong, H.et al (24)	2012	156	PCR	China	Men 135 Women 21	Mean 56	Retrospective research	None	High
Guilemany, J. M.et al (25)	2013	125	ELISA	Spain	Men 80 Women 45	Mean 52.3	Retrospective research	Smoke	High
Pajić Matić, I.et al (26)	2021	77	PCR	Croati	Unknown	Mean 51.3	Prospective research	None	Low
Pirzadeh, A.et al (27)	2011	130	Immunohistochemical evaluation	Iran	Men 126 Women 4	Mean 61.7	Prospective research	Smoke	Low
Rezaii, J.et al (28)	2008	175	ELISA	Iran	Men 147 Women 28	Mean 52	Prospective research	Smoke	High
Rubin, J. S. et al (29)	2003	61	ELISA	England	Men 51 Women 10	Mean 61.5	Prospective research	None	Low
Shi, Y. et al (30)	2011	100	PCR	China	Men 100	Mean 50	Prospective research	Smoke	High
Siupsinskiene, N.et al (31)	2013	83	PCR	Lithuania	Men 57 Women 26	Mean 50.6	Prospective research	Smoke	High
Nurgalieva, Z. Z.et al (32)	2005	230	ELISA	American	Men 127 Women 103	Mean 56	Prospective research	Smoke	High
Burduk, P. K.et al (33)	2013	220	PCR	Poland	Men 65 Women 10	Mean 59.1	Prospective research	None	High
Titiz, A.et al (34)	2008	40	PCR	Turkey	Men 40	Mean 52.4	Prospective research	Smoke	High

A summary of the risk exposures extracted from each study is shown in **Table 2**. The diagnostic methods for Helicobacter pylori infection were different in all studies. The objective measurements listed included PCR and ELISA results and immunohistochemical evaluation. A research based on chart review, used clinical history based on International Classification of Diseases (ICD) codes for diagnosis. **Table 2** showed the diversity of diagnostic methods. Nonsmokers were evaluated in 9 studies. Eight studies conducted multivariate analysis and controlled for patients’ smoking and drinking history.

### Meta-Analysis

**Figure 2:**
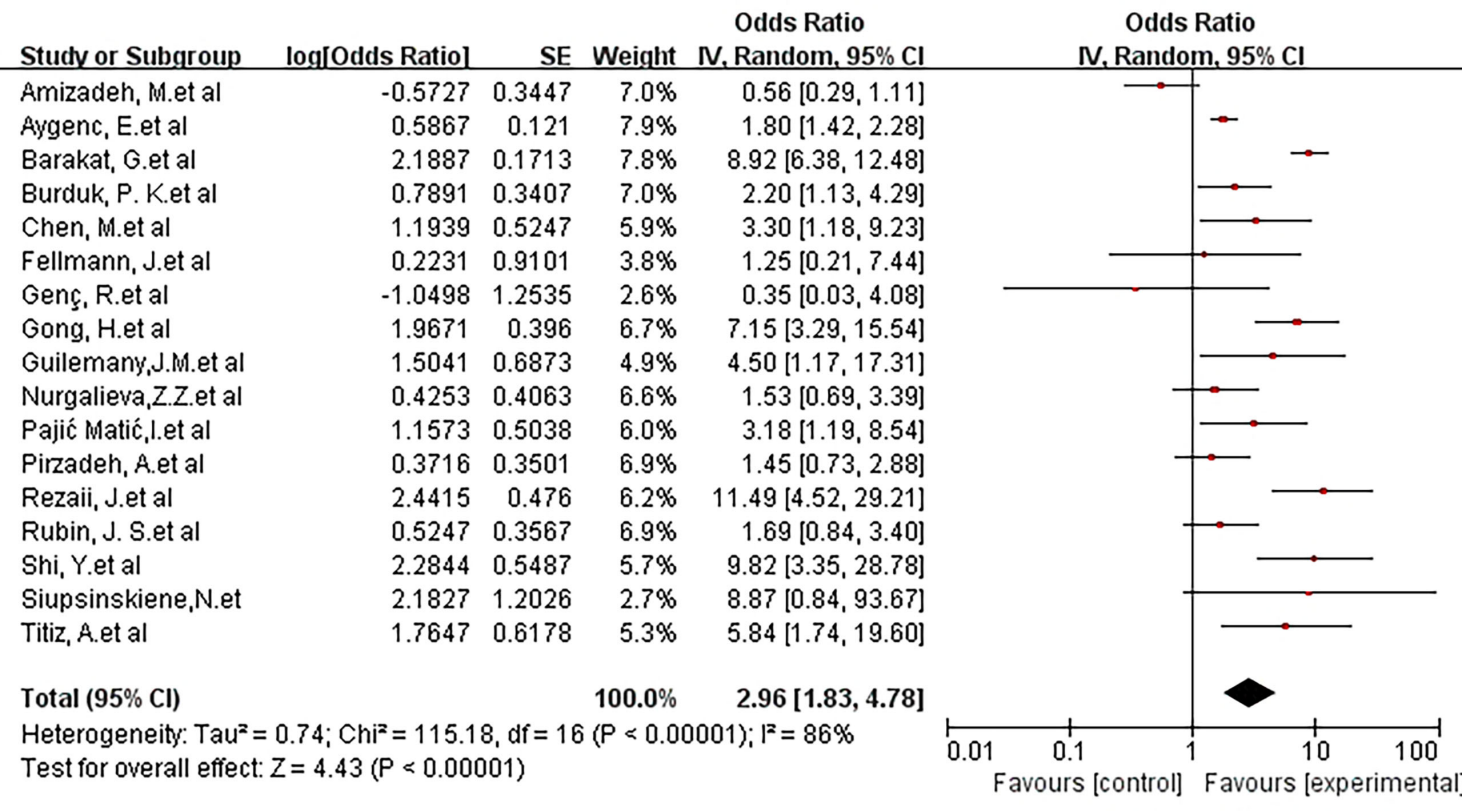
Forest plot showing the overall risk association of *Helicobacter pylori* infection of the larynx and laryngeal cancer.

A random-effects model was selected to conduct a meta-analysis of 17 studies. Overall, the study showed an OR of 2.96(95% CI, 1.83-4.78). The P-value of the overall effect was less than 0.00001, and I^2^ = 86%. This result showed a positive association between Helicobacter pylori infection and Laryngeal cancer risk (**Figure 2**).

### Subgroup Analysis

In the research, we found that the aggregated forest maps were quite heterogeneous (I^2 =^ 86%), so we distinguished several subgroups according to different comparison types. We designed different subgroups to further discuss the reasons for the differences between the groups.The subgroups included diagnostic methods,study design,method quality and smoke exposure.

### Diagnostic Methods

**Figure 3:**
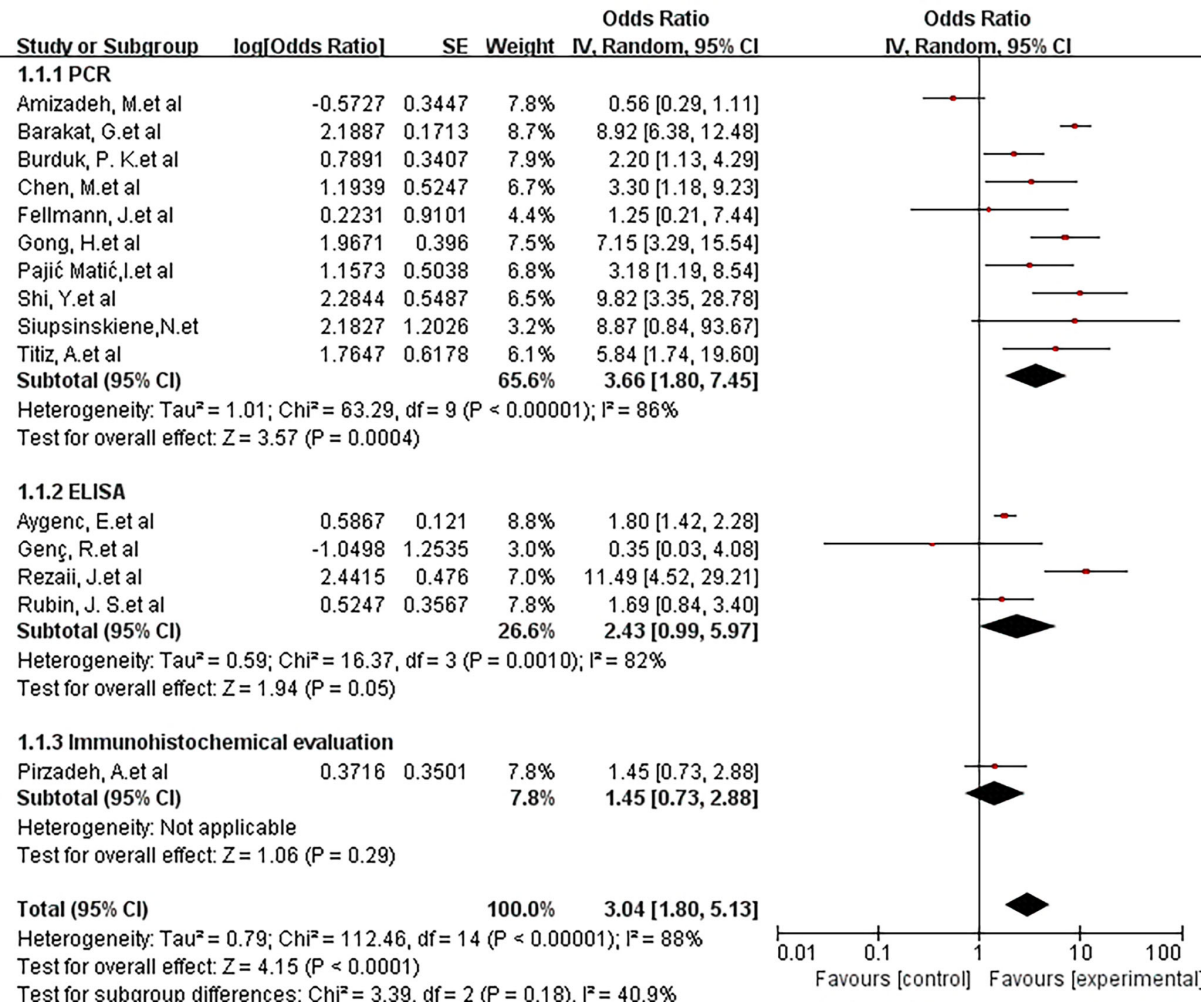
Forest plot showing the overall risk association of Helicobacter pylori infection of the larynx and laryngeal cancer with subgroup analysis based on the type of diagnostic method used.PCR, ELISA, Immunohistochemical evaluation methods are shown. There was no statistically significant difference between the 3 groups. (X^2 =^ 3.39; P=0.18).

The PCR practice was to collect tissue samples from patients diagnosed with laryngeal cancer. Genomic DNA was prepared for polymerase chain reaction. Then through the primer design, followed by several repeated cycles of denaturation - annealing - extension steps, finally electrophoresis detection. ELISA identified helicobacter pylori by gram staining, oxidase, catalase, and urease tests.Immunohistochemical evaluation means that the technologist ranked the density of H. pylori infection according to the number of individual bacteria counted in the high-magnification field (× 1000 light microscope). H. pylori infection density was defined as follows: 0 = 0; 1+ = 1–9; 2+ = 10–29; 3+ = 30–99; and 4+ ≥ 100.A subgroup analysis assessing the type of diagnostic method showed an OR of 3.66 (95% CI, 1.80–7.45; P <0.00001; I^2^ = 86%) for PCR. The OR was 2.43(95% CI, 0.99-5.97; P =0.001; I^2^ = 82%) for ELISA. The OR was 1.45 (95% CI, 0.73–2.88) for the immunohistochemical evaluation method. There were no statistically significant difference between the diagnostic methods.(X^2^ = 3.39; P =0.18; I^2^ = 40.9%) (**Figure 3**).

### Study Design

**Figure 4:**
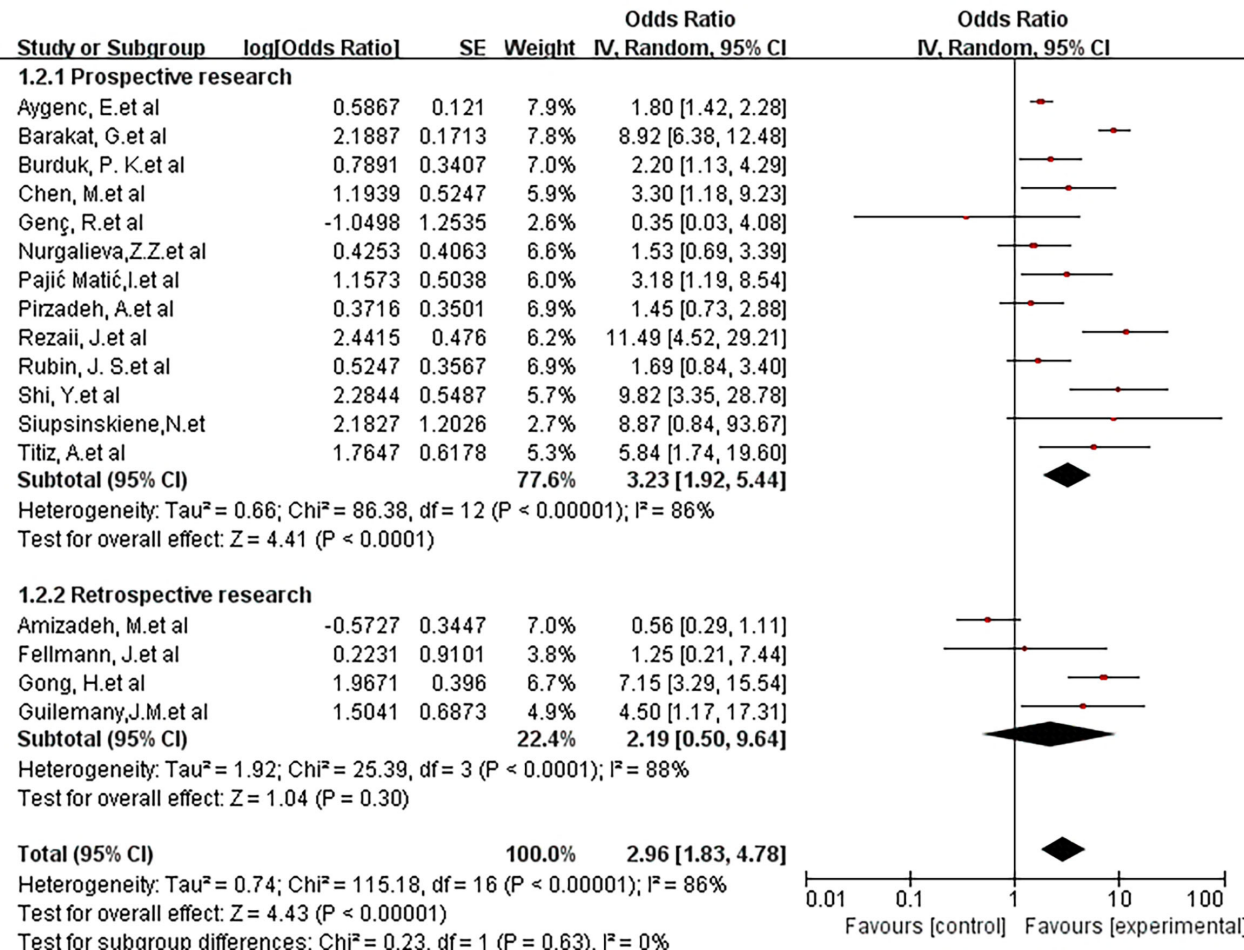
Forest plot showing the overall risk association of *Helicobacter pylori* infection of the larynx and laryngeal cancer with subgroup analysis based on the study method (retrospective vs. prospective).There was no statistically significant difference between the 3 groups. (X^2 =^ 0.23; P=0.63).

A further subgroup analysis was performed to evaluate the impact of the type of study design (prospective or retrospective) on the risk of malignant tumors in the larynx. The combined OR of 13 prospective studies was 3.23(95% CI, 1.92-5.44; P <0.00001; I^2^ = 86%), and the pooled OR of the four retrospective studies was 2.19(95% CI, 0.50-9.64; P <0.0001; I^2^ = 88%). There was no statistically significant difference between the design types (X^2^ = 0.23; P =0.63; I^2^ = 0%) (**Figure 4**).

### Method Quality

**Figure 5:**
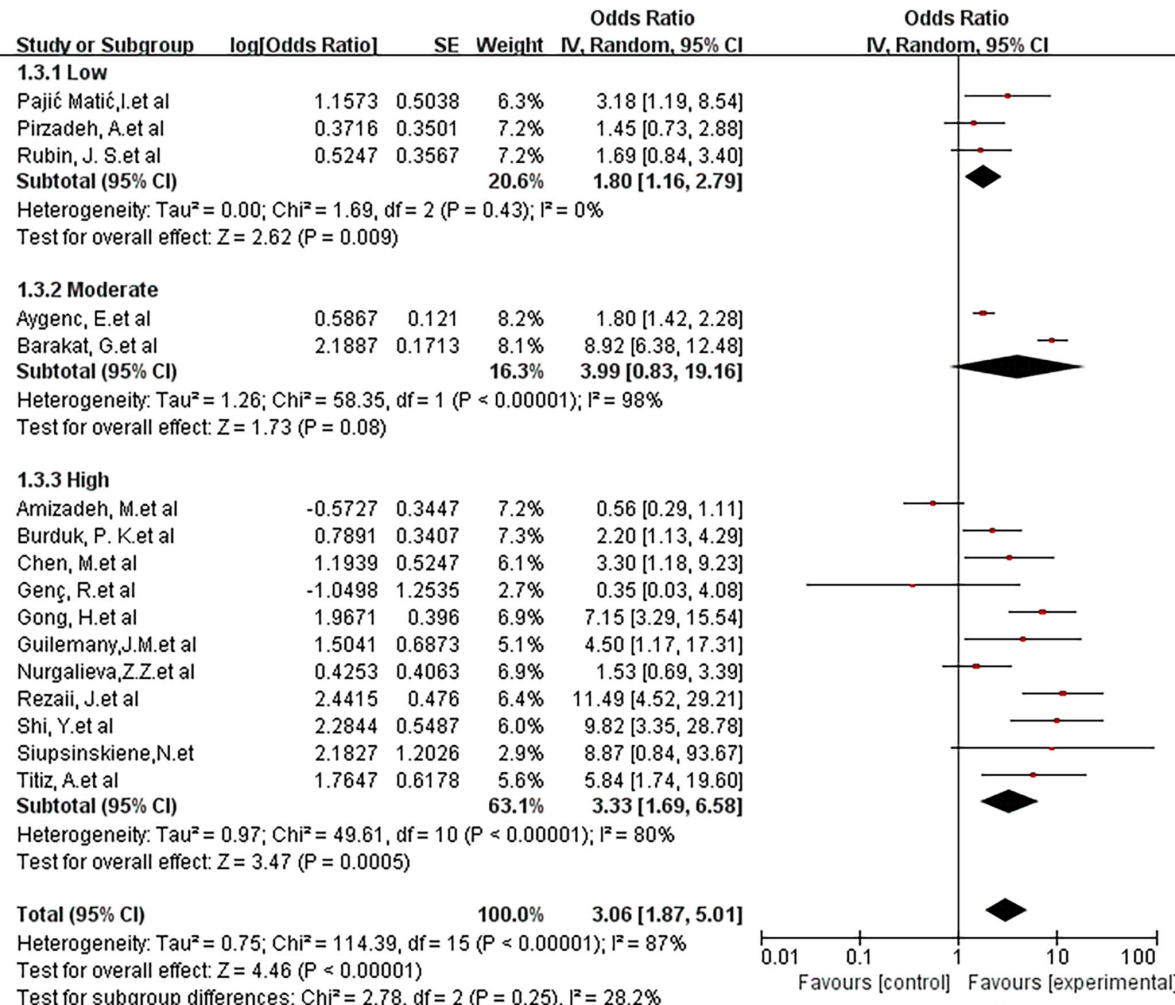
Forest plot showing the overall risk association of *Helicobacter pylori* infection of the larynx and laryngeal cancer with subgroup analysis based on methodologic quality. There was no statistically significant difference between the 3 groups. (X^2 =^ 2.78; P= 0.25).

The influence of methodological quality on risk association was also studied. For this analysis, 4 articles were considered low quality, 2 articles were considered medium quality, and the remaining 11 articles were high quality. Low-quality studies showed an OR of 1.80 (95% CI, 1.16-2.79; P = 0.43; I^2^ = 0%), and medium-quality studies showed an OR of 3.99 (95% CI, 0.83-19.16; P <0.00001; I^2^ = 98%). A high-quality study showed an OR of 3.33 (95% CI, 1.69-6.58; P<0.00001; I^2^ = 80%). There was no statistically significant difference between the 3 groups (X^2^ = 2.78; P =0.25; I^2^ = 28.2%) (**Figure 5**).

### Smoke Exposure

**Figure 6:**
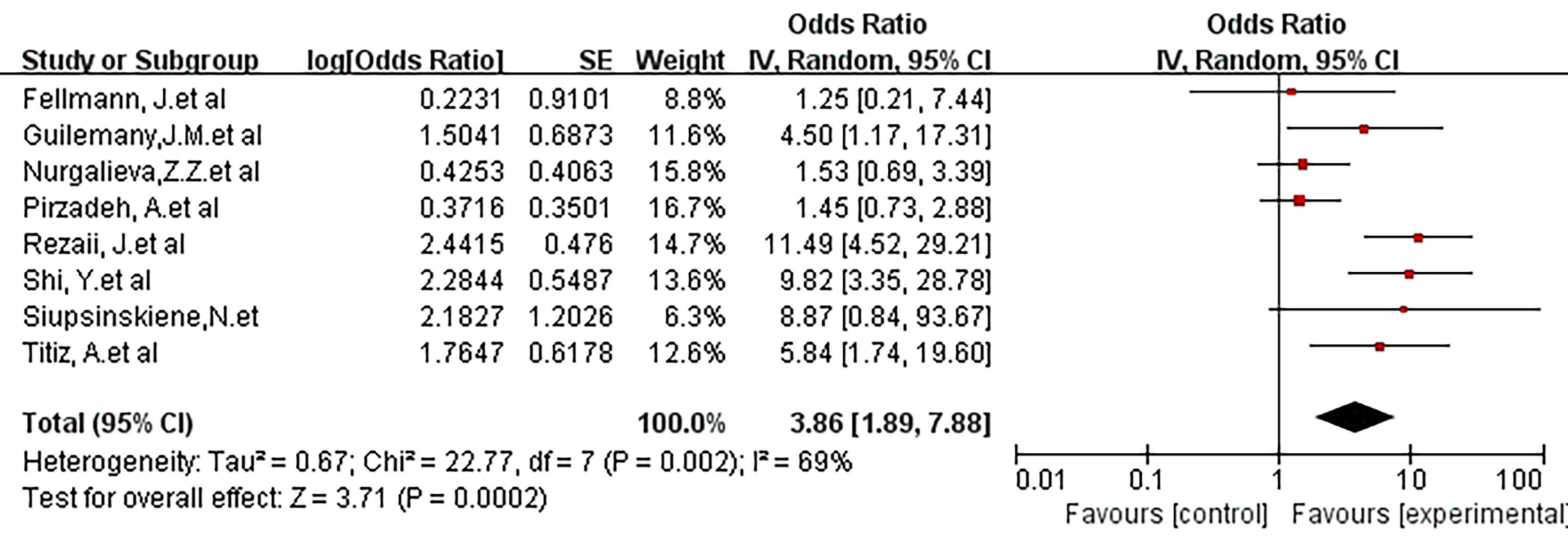
Forest plot showing the risk association of *Helicobacter pylori* infection of the larynx and laryngeal malignancy after controlling for smoking covariates.There was a positive risk association after controlling for smoking (OR, 3.86 [1.86, 7.88]; P =0.0002).

We further discussed the risks of people exposed to smoking. The study of smoking control status showed an OR of 3.86 (95% CI, 1.86-7.88; P =0.0002; I^2^ = 69%). It can be seen that smoking increases the risk of exposure to laryngeal cancer in this part of the population (**Figure 6**).

### Sensitivity Analysis

**Figure 7:**
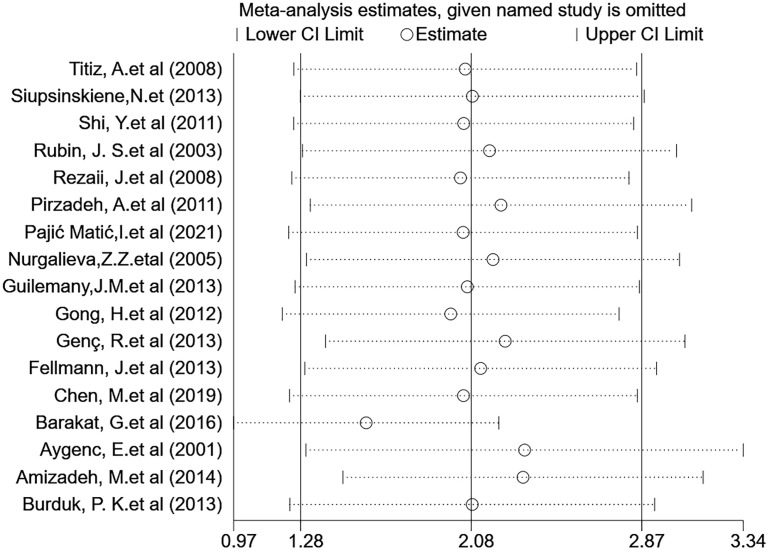
Sensitivity analysis assessing the effect of the included studies on the pooled OR for the combined data.

**Figure 8:**
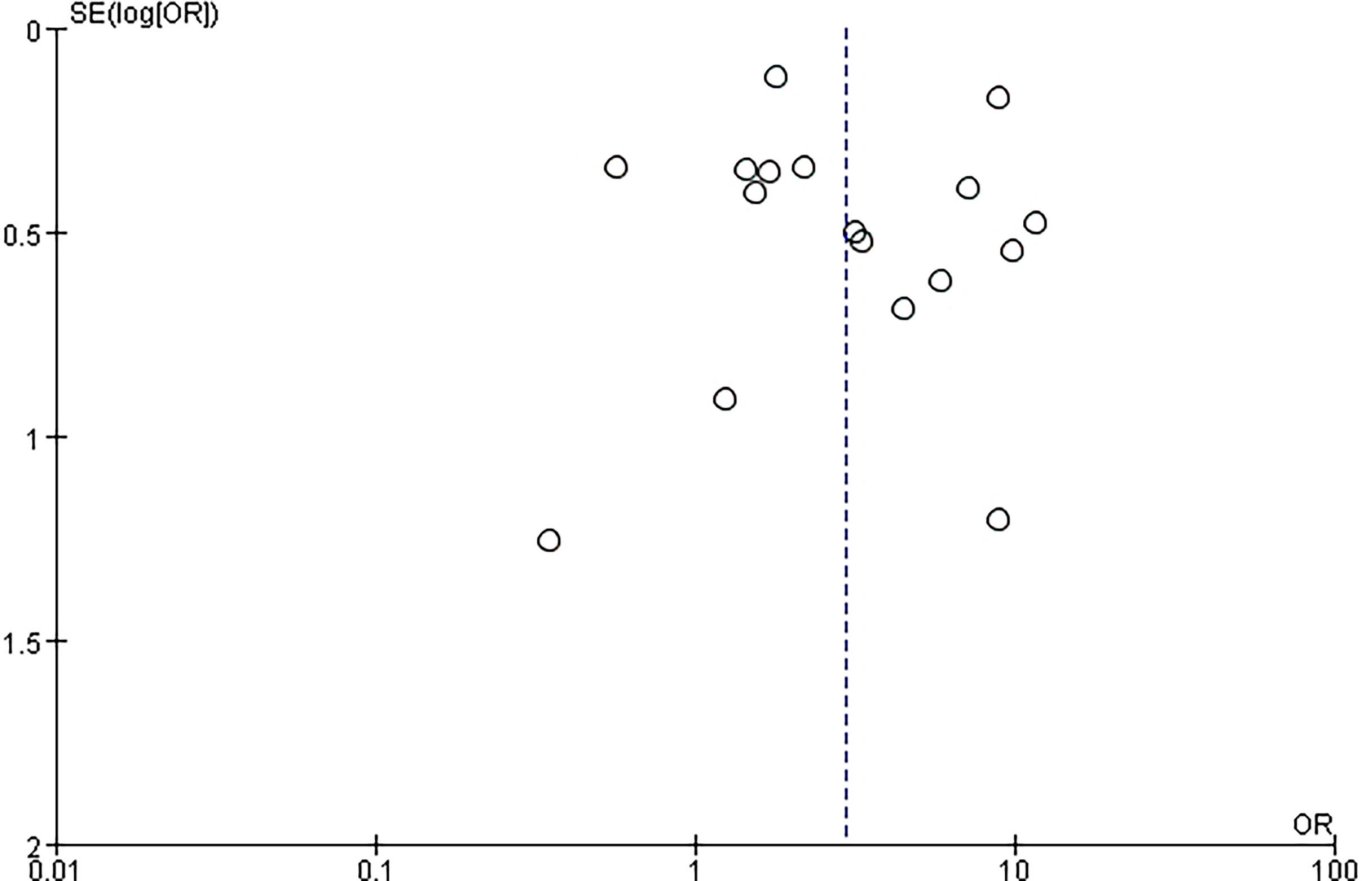
Funnel plot showing the overall risk association of *Helicobacter pylori* infection of the larynx and laryngeal cancer.

Subsequently, a sensitivity analysis was performed to determine the influence of a single study on the OR value of the overall summary data. Excluding individual studies evaluating the OR value after aggregation, the average OR was 2.08, and the average 95% lower and upper limits were 1.28 and 2.87, respectively. Regardless of which study was excluded, the aggregate OR was within an average 95% CI, indicating that no study had a significant impact on the risk association. The funnel graph was slightly asymmetrical (**Figures 7** and **8**).

## Discussion

*Helicobacter pylori* (pylori) is a gram-negative Helicobacter, an important microbial pathogen closely related to the onset of malignant tumors (35). Previous studies reported that the upper respiratory-digestive system was an additional reservoir for a large proportion of H. pylori gastritis patients (16). *Helicobacter pylori* infection was located in the mucosa of the larynx and may be related to vocal cord leukoplakia (18). The oral cavity, tonsil tissue, and saliva were also storage areas for *Helicobacter pylori*. Bacteria can colonize the head and neck area through the oral cavity and the appetite pathway (gastroesophageal reflux) (23). It is of great significance to study the related risk factors for laryngeal cancer and to provide new treatment plans and preventive measures for the occurrence and development of laryngeal cancer. At present, the determination of *Helicobacter pylori* infection is a simple and feasible operation that qPCR can easily detect.

The cytotoxin-associated gene A (CagA) protein of *Helicobacter pylori* can enter human gastric mucosal cells through type IV bacteria. It is an oncogenic protein that can induce tumors in mammals (36). The study by Zhang Q et al. showed that when H. pylori infected humans, it caused fibroblasts and myofibroblasts to transform into cancer-associated fibroblast (CAF)-like cells (37). In addition, their research also suggested that the levels of the proinflammatory factors IL-6, IL-8, COX-2, and SDF-1 increased. Infection can also lead to epithelial-mesenchymal transition in gastric tissue, raising the possibility of gastric cancer development (38). Research by Hong-Li Gong et al. showed that H. pylori infection reduced the expression levels of MSH2 and MLH1 in laryngeal cancer cell lines under coculture conditions. It was suggested that changes in the expression levels of MSH2 and MLH1, a key factor, may affect the overall survival of patients with laryngeal cancer (39). The article by James W T Toh et al. stated that ascorbic acid in gastric juice can reduce the risk of gastric cancer. Ascorbic acid may prevent gastric cancer through its antioxidant effect in gastric cell protection, regenerating active vitamin E and glutathione, reducing the toxic effects of nitrosodimethylamine and heterocyclic amine intake, and preventing Helicobacter pylori infection (40).

The Global Cancer Research Company lists Helicobacter pylori as a type I carcinogen. Different reports indicate that H. pylori has carcinogenic potential in the gastrointestinal mucosa through the interaction between bacterial factors and environmental factors (41). Hadi Maleki Kakelar et al. advocate that the main strategy for treating related cancers is to control the disease with vaccination before the onset of the disease (42). Abolghasem Tohidpour’s research shows that CagA can interact with a variety of proteins in a phosphorylation-dependent and independent manner in the epithelial cells of the digestive tract (43).

The research in this analysis was collected from all current studies on the relationship between *Helicobacter pylori* infection in the larynx and laryngeal cancer. Compared with other studies, the results of this study have certain advantages in terms of accuracy. The subgroups were designed according to the type of study design (prospective or retrospective) on the risk of laryngeal malignancy. The results showed no significant difference between different design types. The subgroup analysis to evaluate the types of diagnostic methods showed that the final OR values of different measurement methods were quite different. Then, subgroups were established according to the influence of method quality on risk association. The results showed that the heterogeneity of the research with low quality grade is 0 after merging, while the heterogeneity of the articles with medium and high grade is still large after merging. We speculated that the reason was caused by the article of Amizadeh and M. Et al. By removing this article, the heterogeneity of the retrospective study group, PCR group and high-quality group were significantly reduced.Finally, a subgroup was designed according to the status of smoking control and the risk of laryngeal cancer. The analysis showed that smoking increases the risk of exposure to laryngeal cancer in this part of the population.This conclusion further supports our point of view.Based on the results of the subgroup analysis, we hypothesized that some low-quality articles had caused more significant heterogeneity. At the same time, the differences caused by the different detection methods may be caused by the improper operation of the experimental materials.

Although the data we obtained indicated an increased association between laryngeal cancer and local infection of the pyloric screw, only a portion of the studies controlled for the influence of factors such as smoking and alcohol consumption. This was not conducive to obtaining real results because these are known risk factors for the occurrence and development of cancer. At the same time, some patients had *Helicobacter pylori* infection in the stomach and the larynx, which can easily cause errors in the control of variables. In addition, we did not find more articles reflecting the relationship between HPV exposure factors and laryngeal cancer. This research can be added to future work.

Esophageal cancer and laryngeal cancer are adjacent to the anatomical location, and both are the process of normal squamous epithelial progression to early invasive cancer. Esophageal cancer and laryngeal cancer are both lesions originating from mucosal epithelium, and most of them are squamous carcinoma, which is sensitive to radiotherapy. Well differentiated squamous cell carcinoma has concentrated nests with intercellular Bridges and carcinoma beads. Poorly differentiated squamous cell carcinoma has no carcinoma beads and is obviously heteromorphic. They all had foreign body sensation in the throat, but for laryngeal cancer, it was evident at quiet time, while for esophageal cancer, it was associated with retrosternal pain during swallowing.Follow-up results of Hyun Lim et al. showed that the 3-year OS rate of HNSCC patients with and without ESCN was 48.2% and 71.2%, respectively. They recommend optimal surveillance to detect early ESCN in HNSCC patients (44). Pepsin is also frequently detected in the larynx of cancer patients, but rarely in patients without clinical symptoms of reflux. Nikki Johnston et al. demonstrated that pepsin increased proliferation of FaDu SCC cells and cultured normal laryngeal epithelial primary cells by increasing the S-phase distribution in flow cytometric analysis in a time - and dose-dependent manner. At the same time, their experiments identified four members of the Let miRNA family as significantly dysregulated in laryngeal epithelial cells exposed to non-acid pepsin. Gastroesophageal reflux is known to cause chemoplastic changes in the esophagus (Barrett’s esophagus) that increase the risk of esophageal adenocarcinoma. The mucosa of the larynx is thought to be more sensitive to the effects of gastric reflux than the esophagus. Therefore, it is reasonable that chronic LPR may lead to tumor changes and promote oncogenic transformation (45).

A recent study showed that GPR12 induces apoptosis by activating caspase-7, and inhibits the migration of hypopharyngeal, laryngeal, and esophageal cancer cells by promoting the expression of EMT-related proteins in hypopharyngeal, laryngeal, and esophageal cancer cells (46). The work of Hala El-Zimaity et al. showed that Helicobacter pylori is associated with esophageal cancer, leading to increased gastric acid secretion. This again intensifies acid reflux, and increases gastroesophageal reflux disease by reducing the pressure of the lower esophageal sphincter (47). Jannis Kountouras et al. reported that HP-I induces gastric microbiota disturbance through sodium hypochlorite and chronic inflammation, which may subsequently affect gerd-Barrett’s esophagus (48). To summarize the above findings, in this part of the population with reflux symptoms, the colonization of hp in the larynx or esophagus will cause mucosal inflammation and promote the development of cancer.

Yen-Ting Lu and others investigated whether patients with peptic ulcer disease have a higher risk of head and neck cancer. Their results showed that peptic ulcer disease was closely related to the increased incidence of laryngeal cancer and hypopharyngeal cancer. *Helicobacter pylori* treatment may help prevent HNC in patients with peptic ulcer disease (49). Whether bacterial infection increases the occurrence of laryngeal cancer or leads to a poor prognosis remains unclear. Whether radical treatment of *Helicobacter pylori* in this part of the population can prolong the survival rate of patients remains unclear. These scientific ideas can enlighten follow-up research work.

## Conclusion

This systematic review and meta-analysis showed an association between Helicobacter pylori and laryngeal cancer. These findings are consistent with the understanding that chronic inflammatory tissue insult may lead to the development of malignancy. Controlling risk factors will help us identify patients with an increased risk of laryngeal cancer.

## Data Availability Statement

The raw data supporting the conclusions of this article will be made available by the authors, without undue reservation.

## Ethics Statement

The study was approved by the Human Research Ethics Committees of The Affiliated Changzhou No. 2. People’s Hospital of Nanjing Medical University, Changzhou, China.

## Author contributions

Conceptualization: YH and MG. Data curation: YH and QW. Formal analysis: YH. Funding acquisition: JL. Investigation: QW and JZ. Project administration: JZ and JW. Software: MG. Supervision: JL and MW. Writing—original draft: YH. Writing—review, and editing: YH and PW. All authors read and approved the final manuscript.

## Funding

This work is supported by Natural Science Foundation of Jiangsu Province(BK20191157), Scientific Program of Changzhou (CJ20190034; ZD201919) and Funding from Young Talent Development Plan of Changzhou Health Commission (2020–233).

## Conflict of Interest

The authors declare that the research was conducted in the absence of any commercial or financial relationships that could be construed as a potential conflict of interest.

## Publisher’s Note

All claims expressed in this article are solely those of the authors and do not necessarily represent those of their affiliated organizations, or those of the publisher, the editors and the reviewers. Any product that may be evaluated in this article, or claim that may be made by its manufacturer, is not guaranteed or endorsed by the publisher.

## Abbreviations

CagA, The cytotoxin-associated gene A; CAF, Cancer-associated fibroblast; IL-6, Interleukin- 6; IL-8, Interleukin- 8; COX-2, Cyclooxygenase-2; SDF-1, Stromal cell-derived factor-1; MSH-2, MutS homolog 2; MLH-1, MutL homolog 1; HNC, Head and neck cancer; GPR12, G protein-coupled receptor 12; LPR, Laryngopharyngeal Reflux; HP-I, Helicobacter pylori infection.
